# Light-Induced Peroxide Formation in ZnO: Origin of Persistent Photoconductivity

**DOI:** 10.1038/srep35148

**Published:** 2016-10-17

**Authors:** Youngho Kang, Ho-Hyun Nahm, Seungwu Han

**Affiliations:** 1 Department of Materials Science and Engineering and Research Institute of Advanced Materials, Seoul National University, 151-742, South Korea; 2 Center for Correlated Electron Systems Institute for Basic Science (IBS) Seoul 151-747, South Korea; 3 Department of Physics and Astronomy, Seoul National University, Seoul 151-747, Korea

## Abstract

The persistent photoconductivity (PPC) in ZnO has been a critical problem in opto-electrical devices employing ZnO such as ultraviolet sensors and thin film transistors for the transparent display. While the metastable state of oxygen vacancy (*V*_O_) is widely accepted as the microscopic origin of PPC, recent experiments on the influence of temperature and oxygen environments are at variance with the *V*_O_ model. In this study, using the density-functional theory calculations, we propose a novel mechanism of PPC that involves the hydrogen-zinc vacancy defect complex (2H-*V*_Zn_). We show that a substantial amount of 2H-*V*_Zn_ can exist during the growth process due to its low formation energy. The light absorption of 2H-*V*_Zn_ leads to the metastable state that is characterized by the formation of 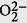 (peroxide) around the defect, leaving the free carriers in the conduction band. Furthermore, we estimate the lifetime of photo-electrons to be ~20 secs, which is similar to the experimental observation. Our model also explains the experimental results showing that PPC is enhanced (suppressed) in oxygen-rich (low-temperature) conditions. By revealing a convincing origin of PPC in ZnO, we expect that the present work will pave the way for optimizing optoelectronic properties of ZnO.

The persistent photoconductivity (PPC) in ZnO wherein the photocurrent is maintained even after light is turned off, is well known for decades^1^,^2^. This phenomenon critically affects the performance of optical and electrical devices utilizing ZnO, mostly in negative ways. For example, in the application such as ultraviolet sensor, PPC in ZnO undermines the reliability and response speed of the photodetector^3^,^4^. As another example, thin-film transistor for next-generation displays utilizing ZnO-based oxide semiconductors suffers from the light instability issues that correlate with PPC^5^,^6^,^7^. Therefore, understanding PPC is critical to overcome the material issues and expand the application area of ZnO.

The microscopic origin of PPC in ZnO has been studied intensively in numerous literatures and several models were proposed^8^,^9^,^10^,^11^,^12^. As an extrinsic mechanism, it was suggested that the desorption of surface oxygen molecules upon light illumination plays a key role in controlling the lifetime of electron carriers^10^,^12^,^13^,^14^. This mechanism also explains the extremely long PPC of ZnO nanowire in vacuum^12^. In addition, several experimental results further confirmed this model by showing that the surface passivation by organic molecules or other thermal oxide suppresses PPC. However, the significant level of PPC still remains even with the surface passivation, implying that the intrinsic origin also exists^10^,^14^.

It is widely accepted that the metastable conducting state of oxygen vacancy (*V*_O_) constitutes a major intrinsic source for PPC in ZnO, which was first suggested theoretically^9^. In this model, electrons trapped at *V*_O_ are excited into the conduction band upon light absorption, resulting in the doubly ionized *V*_O_ (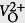). Concurrently, neighboring Zn ions undergo a large outward relaxation and the empty level of *V*_O_ shifts into the conduction band, impeding recombination of photo-carriers and defect levels of 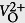 9. However, the previous calculation showed such metastablility of 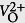 is rather short-lived due to the small energy barrier against recombination^11^. The vacancy model was complemented in a recent study^8^ wherein the electrons trapped at the hydrogen-*V*_O_ complex are released upon light illumination and then fall back into the defect site with an energy barrier of ~0.36 eV.

While many experiments on PPC support the *V*_O_ mechanism^15^,^16^,^17^, several experimental results are at odds with the *V*_O_ model. For example, PPC was not decreased^18^ or even increased^19^ when the oxygen partial pressure was increased during the deposition of ZnO film. These are at variance with the *V*_O_ model because the density of *V*_O_ should be reduced at higher oxygen pressure. In addition, PPC was found to be significantly suppressed at ~70 K in comparison with that at room temperature^20^. This implies that the metastable conducting state forms with a small but finite activation energy. However, theoretical calculations on 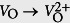 transition preclude any thermal barrier and the metastable conducting state forms spontaneously when electrons are excited into the conduction band under illumination^9^. One may expect that the low equilibrium density of *V*_O_ is responsible for the suppression of PPC at low temperatures. However, we note that the temperatures in Ref. 20 referred to those for the measurement of PPC, not for growth or annealing processes, and it would be hard for *V*_O_ to equilibrate promptly during measurement, particularly at low temperatures. Therefore, we conclude that the *V*_O_ models are not satisfactory in explaining PPC in its full picture.

In this study, using the first-principles calculations, we suggest a new mechanism for PPC based on hydrogen-zinc vacancy defect complex or *N*H-*V*_Zn_, where *N* indicates the number of hydrogen. We reveal that light absorption at 2H-*V*_Zn_ results in O-O bond that gives rise to the metastable conducting states. The formation of O-O bond and recovering to the original state of 2H-*V*_Zn_ requires energy barriers of ~0.45 eV, to be consistent with experiment.

## Results and Discussions

The concentration of a defect in the host material relies on the amount of available sites to form the defect and its defect formation energy (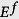) that depends on growing or annealing conditions. In addition, if a defect is charged, 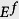
is affected by Fermi level (*E*_F_) corresponding to the chemical potential of an electron. 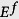
of individual defect in ZnO can be evaluated using the total energies of DFT calculation as follows^9^,^11^.


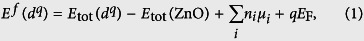


where *E*_tot_(*d*_^q^_) and *E*_tot_(ZnO) are total energies of a supercell including a defect with charge *q* (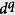) and ZnO perfect crystal. In Equation (1), *n*_*i*_ is the number of *i*^th^ element added into or removed from ZnO and *μ*_*i*_ is its chemical potential that depends on growth conditions. The upper bounds of *μ*_Zn_ and *μ*_O_ are set to the energy of Zn metal [*E*_tot_(Zn)] and a half of oxygen molecule [*E*_tot_(O_2_)/2], which corresponds to Zn-rich and O-rich conditions, respectively. The thermodynamic stability condition for ZnO gives rise to the lower bound of *μ*_Zn_ in O-rich condition: *μ*_Zn_ = *E*_tot_(Zn) + Δ*H*_*f*_(ZnO) where Δ*H*_*f*_(ZnO) is the heat of formation of ZnO. Conversely, the lower limit of *μ*_O_ in Zn-rich condition is *μ*_O_ = *E*_tot_(O_2_)/2 + Δ*H*_*f*_(ZnO). We use the chemical potential of hydrogen (*μ*_H_), as *μ*_H_ = *E*_tot_(H_2_)/2 assuming the H-rich condition.

**Figure 1:**
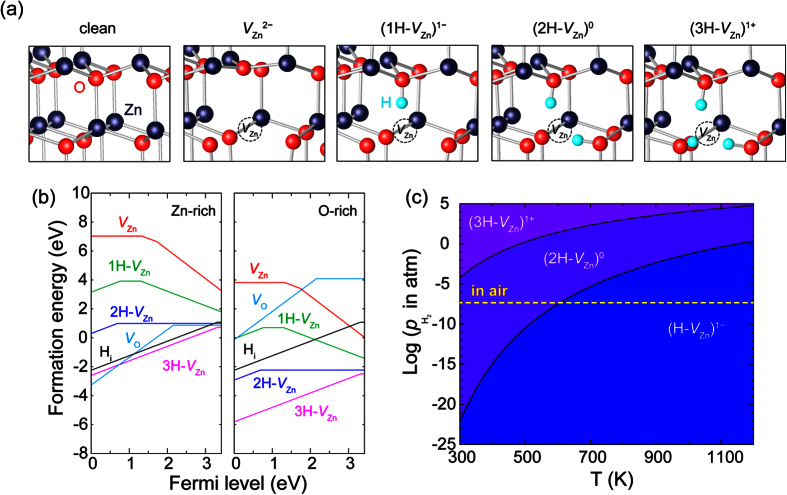
(**a**) Atomic structures of ZnO without defects, 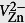, (1H-*V*_Zn_)^1−^, (2H-*V*_Zn_)^0^, and (3H-*V*_Zn_)^1+^. (**b**) Defect formation energies with respect to the Fermi level for Zn-rich and O-rich limit. (**c**) The phase diagram of *N*H-*V*_Zn_ depending on hydrogen partial pressure and temperature for *E*_F_ = *E*_*c*_. The yellow line is the partial pressure of H_2_ gas in air.

We calculated 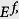
s for *V*_O_, *V*_Zn_, hydrogen interstitial (H_i_) and *N*H-*V*_Zn_ at 0 K. We considered H_i_ at a bond-centered position along the *c*-axis which is known to be the energetically favorable site^21^. In *N*H-*V*_Zn_, H atoms form chemical bonds with the O atoms near to the *V*_Zn_ site. There are four O sites available for the formation of O-H bond as shown in Fig. 1(a). In this study, we only present data for *N*H-*V*_Zn_ with *N* = 1, 2, and 3, as 4H-*V*_Zn_ is unlikely due to the large formation energy. For *N*H-*V*_Zn_, two different configurations of *N*H-*V*_Zn_ are possible for each *N* depending whether one O-H bond is parallel to the *c*-axis of wurtzite structure (OH_∥_). In this study, we always include the OH_∥_ which was confirmed by the previous experiments for *N* = 2 via analyzing infra-red absorption spectrum^22^,^23^,^24^. (The two configurations differ in energy only by ~0.1 eV, to be consistent with previous literature^25^).

The computed 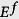
’s as a function of *E*_F_ are shown in Fig. 1(b) in which the valence band maximum, *E*_*v*_, is set to 0 and the upper limit of *E*_F_ corresponds to the conduction band minimum, *E*_*c*_. The Fermi level at which the slope is changed from *q* to *q*′ is called the charge transition level, *ε*(*q*/*q*′). *V*_Zn_ is a double acceptor and its charge state is always 2− in the *n*-type condition (*E*_F_~*E*_*c*_). In contrast, H_i_ is a shallow donor with *ε*(1+/0)~*E*_*c*_ and it is regarded as an origin for the unintentional electron doping in ZnO. The electrical state and 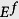
of *N*H-*V*_Zn_ are dependent upon *N* in the *n*-type material; 1H-*V*_Zn_ exists in the negative charge state capturing electron carriers in the conduction band of the host material. In contrast, the neutral charge state is the most stable in 2H-*V*_Zn_ and so it is electrically inactive. Further hydration of 2H-*V*_Zn_ results in 3H-*V*_Zn_ that acts as a shallow donor with *ε*(1+/0) close to *E*_*c*_. The 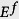
’s of *N*H-*V*_Zn_ are substantially smaller than the sum of those of isolated defects, reflecting strong binding between H and *V*_Zn_. We note that the 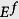
’s of *N*H-*V*_Zn_ are comparable to that of *V*_O_ even in Zn-rich condition implying that these defect pairs may occur in modest concentrations in ZnO regardless of growing conditions.

So far, we discussed the extreme case of H-rich condition at 0 K, but in the real system, the chemical potential of hydrogen in gas phase substantially depends on the temperature, *T*, and its pressure, *p*_H2_, as follows^26^


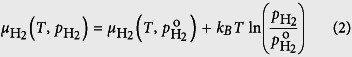



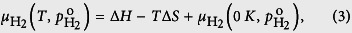


where 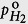 and *k*_*B*_ are the pressure at the standard state and Boltzmann constant. Δ*H* and Δ*S* indicate the enthalpy and entropy changes between 0 K and *T* at 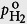, respectively, which can be obtained from the thermochemical table^27^. 
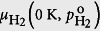
 in Equation (3) is equal to *E*_tot_(H_2_).

Figure 1(c) shows the most stable configuration of (*N*H-*V*_Zn_) in 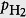-*T* space for *E*_F_ = *E*_*c*_. i.e., *n*-type condition. *N* in stable complex defect tends to be reduced with increase(decrease) of *T*(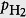). (2H-*V*_Zn_)^0^ is found to be stable over a wide range of 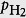 and *T* including the ambient condition. This implies that (2H-*V*_Zn_)^0^ is easily formed by uptake of hydrogen in an atmosphere at various experimental conditions. In addition, the formation of (2H-*V*_Zn_)^0^ would be kinetically feasible due to the highly mobile 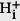 and Coulomb attraction between 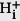 and the anionic defects such as (1H-*V*_Zn_)^1−^ and 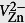. Therefore, the unintentional H source in the forming gas can enlarge the concentration of (2H-*V*_Zn_)^0^ during growth and annealing^21^. Even though annealing in the ultra-high vacuum or at the high temperature can transform (2H-*V*_Zn_)^0^ into (1H-*V*_Zn_)^1−^, we assume that (2H-*V*_Zn_)^0^ is kinetically stable because dissociation of H from the defect complex requires a large energy barrier. Specifically, our calculation yields more than 2 eV for the dissociation energy of hydrogen in (2H-V_Zn_)^0^ → (1H-*V*_Zn_)^1−^+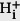, to be consistent with the previous calculation^24^. Indeed, the signal of the local vibrational mode of (2H-*V*_Zn_)^0^ remained up to 600 °C in Ar environment^24^. In addition, we considered H_2_O as a limiting phase to examine whether hydrogen can remain in ZnO, rather than forms water, during the synthesis in oxygen-rich environments. It is found that (2H-*V*_Zn_)^0^ is still stable with the formation energy of 0.69 eV, and therefore our conclusion in the below would not be affected.

**Figure 2:**
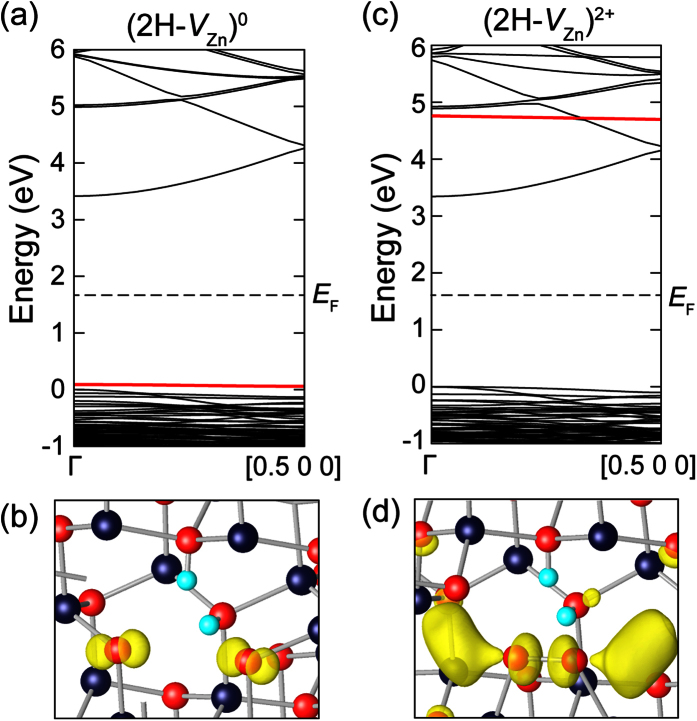
(**a,c**) The band structures of (2H-*V*_Zn_)^0^ without the peroxide and (2H-*V*_Zn_)^2+^ with the peroxide, respectively. The localized levels developed by non-bonding states of oxygen atoms in (2H-*V*_Zn_)^0^ and the anti-bonding state of peroxide in (2H-*V*_Zn_)^2+^ are colored in red. (**b**) and (**d**) show the charge-density distributions corresponding to the defect levels (red) in, (**a**) and (**c**), respectively. The values of isosurfaces are 0.016 e/Å^3^ for (**b**) and 0.005 e/Å^3^ for (**d**).

Figure 2(a) shows the band structure of ZnO in the presence of (2H-*V*_Zn_)^0^. It is seen that localized states develop above the valence top (see the energy level in red). This state originates from the lone pair of two non-bonding oxygen atoms. This is confirmed by the isosurface of charge density of the corresponding state in Fig. 2(b). The two electrons occupying this state are donated by H atoms.

The light illumination on the defect complex will excite the electrons trapped at the non-bonding pair to the conduction band, changing the charge state to (2H-*V*_Zn_)^2+^. As a result, the electronic configuration of non-bonding oxygen changes from O^2−^ in (2H-*V*_Zn_)^0^ to O^1−^ in (2H-*V*_Zn_)^2+^. Since the octet rule is not satisfied with O^1−^, a covalent bond forms between adjacent O^1−^ ions, resulting in a peroxide (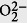):


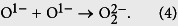


The peroxide formation reduces the total energy by 0.48 eV compared to 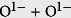 configuration. Figure 2(c) shows the band structure in the presence of peroxide. It is seen that the empty state shifts up into the conduction band (see the energy level in red). This is because the oxygen *p* orbitals in 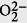 overlap significantly and result in a large level splitting between bonding and anti-bonding states^28^. The charge density of the empty level in Fig. 2(d) clearly shows that the direction of *p* orbital and nodal structure correspond to *pp*σ* bonding. Since there is no empty state in the band gap that can capture the photo-electrons in conduction band, (2H-*V*_Zn_)^2+^ with peroxide results in the metastable conducting state. This will be discussed in detail in the below. (We also examined the metastable conducting state of (2H-*V*_Zn_)^+2^ without OH_∥_ and we found that the energy of 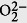 is still smaller than O^1−^ + O^1−^ configuration by 0.38 eV and *pp*σ* state appears above the conduction band minimum. Therefore, the detailed mechanism of PPC we discuss below does not depend on the specific configurations of (2H-*V*_Zn_)^2+^).

**Figure 3:**
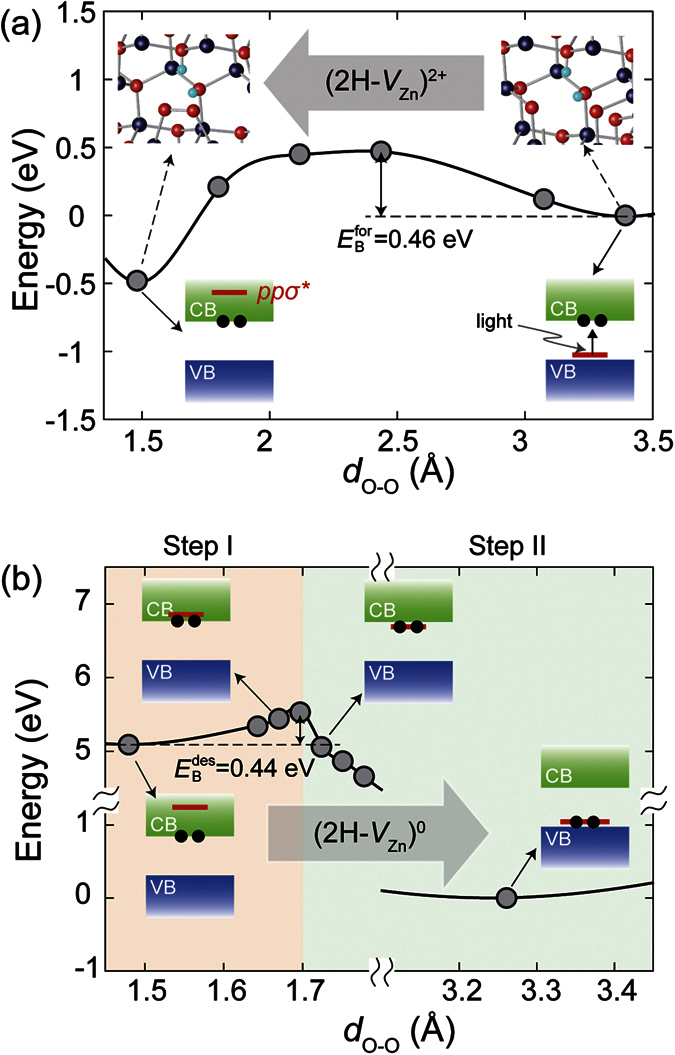
The energy profile for (**a**) the formation of the peroxide in (2H-*V*_Zn_)^2+^ and (**b**) the destruction of the peroxide in (2H-*V*_Zn_)^0^ as a function of *d*_O-O_. 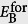 and 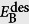 are the energy barriers for the formation and destruction of the peroxide, respectively, when 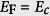. In (**a**), we present the atomic configurations at the corresponding points in the diagram. In (**b**), Step I is the energy profile when the photo-electrons lie at the conduction bottom and Step II is the relaxation energy curve after 2H-*V*_Zn_ state traps the photo-electrons. The band structures show the position of the trap level (red line) and photo-electrons (black filled circles) at a given *d*_O-O_.

To investigate the kinetics of peroxide formation and destruction in 2H-*V*_Zn_, we calculate the energy barriers in Fig. 3. Here, we used the constrained optimization method in calculating the energy barriers. That is to say, the positions of all atoms are relaxed while the distance between two non-bonding oxygen atoms is varied with specific values. We confirmed that the discrepancy with more advanced method such as the nudged elastic band method^29^ is less than 2%. Figure 3(a) shows the formation energy of (2H-*V*_Zn_)^2+^ as a function of the distance between the two oxygen atoms (*d*_O-O_) forming the peroxide. The Fermi level is set to *E*_*c*_, meaning that the two electrons liberated by photons stay at the conduction minimum. Although the peroxide state is energetically favorable by 0.48 eV, its formation requires an energy barrier (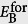) of 0.46 eV due to the loss of Zn-O bonds of the non-bonding oxygen atoms before forming peroxide. Within the transition state theory, the transition time is ~5 μs at room temperature assuming the attempt frequency of 10 THz, a typical frequency of atomic vibration in oxides^30^. At low temperatures of 70 K, however, the transition time is practically infinite, implying that the peroxide formation is mostly suppressed and the metastable conducting state or PPC do not occur. This is consistent with experiment^20^.

Within the present model, the lifetime of photocurrent relies on the transition time for the photo-excited electrons to be trapped at 2H-*V*_Zn_, destructing the peroxide. In the above it was shown that the peroxide formation shifts the *pp*σ* level well into the conduction band. This means that the decay of free electrons proceeds through two steps as shown in Fig. 3(b); in Step I, the *pp*σ* level is lowered below *E*_*c*_ when *d*_O-O_ is elongated due to the thermal vibration of peroxide. In Step II, the free electrons fall into this level, which will spontaneously relax the structure into (2H-*V*_Zn_)^0^.

It is seen in Fig. 3(b) that the energy barrier for the *pp*σ* level to cross *E*_*c*_ (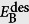) is 0.44 eV which corresponds to the energy required for the peroxide bond to be elongated sufficiently. The average decay time of photo-electrons (*τ*_d_) can be estimated by considering the shift of the *pp*σ* level down to *E*_*c*_ and the entailing recombination of photo-electrons into this level. We suggest that the concentration of photo-electron (*n*_pe_) satisfies the following equation.


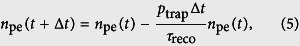


where *p*_trap_ is the probability for the *pp*σ* level to lie below *E*_c_ during Δ*t*. That is to say, *p*_trap_Δ*t* is the time window that allows for the decay of photo-electron. Because the downshift of *pp*σ* results from thermal vibration of O-O bonding in peroxide, 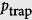 should follow the Arrehnius form of 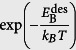. In Equation (5), 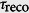 is the recombination time of photo-electron and holes at *pp*σ* level and we assume a constant value of 1 μs that corresponds to the luminescence lifetime for the sub-gap emission in ZnO^31^. For the infinitesimally small Δ*t*, the solution of Equation (5) is given as follows:


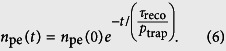


Therefore,


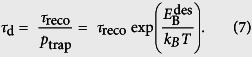


At 300 K, the Equation (7) results in ~20 sec for 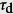, showing good agreement with the experiment by Murphy *et al.*, which reported 55 sec of *τ*_d_ for ZnO film with SiO_2_ passivation layer and carrier concentration of ~10^18^ cm^−3^ 10. We note that *τ*_d_ is much longer than the formation time of peroxide (~5 μs) although the energetic barrier is almost the same (0.46 versus 0.44 eV). This is because the destruction of peroxide requires additional electronic transition from the conducting state to the *pp*σ* level.

In the introduction, we mentioned the inconsistencies between current *V*_O_ models and experimental results. The present model is consistent with these experiments: first, our result indicates that PPC is thermally activated due to the energy barrier for the formation of 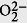, which is supported by the experiment showing the suppression of PPC at low temperatures^19^. Furthermore, our model based on (2H-*V*_Zn_)^0^ defect is consistent with the enhancement of PPC for ZnO grown under oxygen rich condition^18^,^19^ because the density of (2H-*V*_Zn_)^0^ increases in this condition. Lastly, we note that PPC can be reduced by further hydrogenation of (2H-*V*_Zn_)^0^ which leads to the formation of (3H-*V*_Zn_)^1+^; indeed, the reduction of PPC was found in ZnO film grown in ultrahigh purity of H_2_ 18.

## Conclusion

In conclusion, we proposed a new microscopic model for PPC mechanism in ZnO based on the defect complex of (2H-*V*_Zn_)^0^. Upon the light absorption, peroxide is formed within the defect complex, which shifts the empty level well into the conduction band and gives rise to PPC. The recombination of photo-electrons with holes requires a thermal energy of 0.45 eV, which results in the average decay time of ~20 secs. The present model can explain several experiments reporting different PPC behaviors with respect to growth environments. The peroxide has not been reported experimentally yet and its verification would be challenging because peroxide is a metastable state. Nevertheless, it might be possible to detect the formation of peroxide as its lifetime is substantially long once they are created. Finally, PPC is commonly observed in oxide semiconductors like In_2_O_3_ and SnO_2_ that have similar electrical properties with ZnO 32,33. Therefore, we believe that the results of this work can be extended to explain PPC in other semiconducting oxides as well.

## Methods

All the calculations in this study were carried out using Vienna *ab initio* simulation package (VASP)^34^. The projector-augmented wave (PAW) pseudopotential is employed for the ionic potential^35^ with 500 eV of cutoff energy for the plane wave basis set and hybrid functional method based on HSE06 is used for the exchange-correlation energy^36^. The fraction of exact exchange energy we used is 0.372 which gives experimental band gap of 3.42  eV of ZnO. We used 0.05 eV/Å as the stopping criteria for relaxation. The calculated lattice parameters of ZnO are *a* = 3.24 Å and *c* = 5.22 Å which are consistent with experimental values within 1% error^37^.

For the defect calculations, 4 × 4 × 3 supercell with 192 atoms is employed and only the Γ point is sampled. The spin-polarized calculation was performed regardless of the number of electrons in the system in order to take into account the localized nature of oxygen *p* orbitals. For example, the ground state of 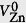 in ZnO is confirmed to be ferromagnetic state in which two holes are localized in oxygen non-bonding orbital, separately, and it is energetically more favorable than non-magnetic state^38^. In the case of charged defects, the spurious interaction between repeated images is removed by monopole correction together with potential alignment^39^. The 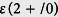 of *V*_O_ is found to be *E*_*v*_+2.1 eV which is consistent with previous hybrid functional calculation^40^. For the calculations of the HSE band structures for the supercells with (2H-*V*_Zn_)^0^ and (2H-*V*_Zn_)^2+^, we calculated the energy eigenvalues from Γ to [0.5 0 0] with only the weight of the Γ point to be 1. In addition, since the ground states of these defects are non-magnetic, we carried out the spin-unpolarized calculations in plotting the band structure.

## Additional Information

**How to cite this article**: Kang, Y. *et al.* Light-Induced Peroxide Formation in ZnO: Origin of Persistent Photoconductivity. *Sci. Rep.* **6**, 35148; doi: 10.1038/srep35148 (2016).
